# Clinical and Ultrasonographic Evaluation of Lower-extremity Vein Thrombosis in Behcet Syndrome: An Observational Study

**DOI:** 10.1097/MD.0000000000001899

**Published:** 2015-11-06

**Authors:** Emire Seyahi, Osman Serdal Cakmak, Burcin Tutar, Caner Arslan, Atilla Suleyman Dikici, Necdet Sut, Fatih Kantarci, Hasan Tuzun, Melike Melikoglu, Hasan Yazici

**Affiliations:** From the Division of Rheumatology, Department of Medicine (ES, MM, HY); Department of Medicine (OSC); Department of Radiology (BT, ASD, FK); Department of Cardiovascular Surgery, Cerrahpaşa Medical Faculty, University of Istanbul, Istanbul (CA, HT); and Department of Biostatistics, Medical Faculty, University of Trakya, Edirne, Turkey (NS).

## Abstract

Vascular involvement can be seen in up to 40% of patients with Behcet syndrome (BS), the lower-extremity vein thrombosis (LEVT) being the most common type. The aim of the current study was to compare venous Doppler findings and clinical features between BS patients with LEVT and control patients diagnosed as having LEVT due to other causes.

All consecutive 78 patients (71 men, 7 women; mean age 38.6 ± 10.3 years) with LEVT due to BS and 50 control patients (29 men, 21 women; mean age 42.0 ± 12.5 years) who had LEVT due to other causes, or idiopathic, were studied with the help of a Doppler ultrasonography after a detailed clinical examination. Patterns of venous disease were identified by cluster analyses. Clinical features of chronic venous disease were assessed using 2 classification systems. Venous claudication was also assessed.

Patients with BS were more likely to be men, had significantly earlier age of onset of thrombosis, and were treated mainly with immunosuppressives and less frequently with anticoagulants. Furthermore, they had significantly more bilateral involvement, less complete recanalization, and more frequent collateral formation. While control patients had a disorganized pattern of venous involvement, BS patients had a contiguous and symmetric pattern, involving all deep and superficial veins of the lower extremities, with less affinity for crural veins. Clinical assessment, as measured by the 2 classification systems, also indicated a more severe disease among the BS patients. In line, 51% of the BS patients suffered from severe post-thrombotic syndrome (PTS) and 32% from venous claudication, whereas these were present in 8% and 12%, respectively, among the controls. Among BS patients, a longer duration of thrombosis, bilateral femoral vein involvement, and using no anticoagulation along with immunosuppressive treatment when first diagnosed were found to be associated independently with severe PTS.

Lower-extremity vein thrombosis associated with BS, when compared to LEVT due to other causes, had distinctive demographic and ultrasonographic characteristics, and had clinically a more severe disease course.

## INTRODUCTION

Vascular involvement can be seen in up to 40% of patients with Behcet syndrome (BS).^1^ Lower-extremity vein thrombosis (LEVT) is the most common type of involvement.^1–4^ It generally starts within few years of disease onset and tends to precede major vessel involvement at other sites. Similar to the other manifestations of BS, it runs a relapsing course. Apart from 2 previous studies from our group,^5,6^detailed Doppler ultrasonographic evaluation of LEVT, along with its clinical associations, has not been available.

The post-thrombotic syndrome (PTS) is a common chronic complication of LEVT with chronic pain, leg edema, and stasis ulcerations.^7–10^ We had the impression that LEVT due to BS had a more severe course, resulting in a higher frequency of PTS than is the case with garden-variety LEVT. This comparison has not formally been made.

Thus, the objective of the current study among BS patients with known LEVT, along with control patients diagnosed as having LEVT due to other causes, was to compare the venous Doppler ultrasonography (USG) findings and their association with the clinical course between the 2 groups, with special emphasis on development of PTS.

## PATIENTS AND METHODS

We studied consecutive BS patients with LEVT who attended the dedicated BS outpatient clinic at the Cerrahpaşa Medical Faculty of University of Istanbul, and control patients who had LEVT due to other causes who attended the outpatient clinic of the Department of Cardiovascular Surgery of the same institution. Both patients and controls were consecutive outpatients seen within the same time period. All BS patients had fulfilled the International Study Group Criteria.^11^ BS signs and symptoms were sought in all controls, and only those patients who did not meet the criteria for BS were included in the study. All patients in the control group underwent a detailed investigation for thrombophilia; however, such an investigation was not done in the routine work-up for BS.

By definition, all patients and the controls had a documented thrombotic event in the lower extremities. LEVT was defined as any (acute, subacute, or chronic) thrombotic event in any (superficial or deep) lower-extremity vein demonstrated by Doppler USG. The veins involved as documented on the first available Doppler USG were also recorded. A few patients with only venous insufficiency from either group were excluded.

### Lower-extremity Doppler USG Assessment

All patients and controls were reanalyzed using color Doppler USG in the Radiology Department, by a radiologist who was blinded to the clinical diagnosis of the patients and controls. In each patient, a total of 16 superficial and deep veins in both legs were assessed for the presence or absence of obstruction, recanalization, reflux, and collaterals. These were as follows: iliac vein, common femoral vein (CFV), deep femoral vein (DFV), superficial femoral vein (SFV), popliteal vein (PV), vena saphena magna (VSM), vena saphena parva (VSP), and crural veins. USG examinations were completed within 2 weeks following the clinical examination.

### Clinical Assessment

The age at first thrombotic event was obtained from the charts. Demographic and clinical characteristics, drugs used after the diagnosis of thrombosis and those currently used, along with smoking and working status, were surveyed by a standardized questionnaire (Appendix 1, http://links.lww.com/MD/A482). Clinical features of chronic venous disease were graded by the same physician (OSC) according to 2 classification systems, both developed by the American Venous Forum (Appendix 1, http://links.lww.com/MD/A482).^7^ The first was the descriptive clinical, etiologic, anatomical, and by pathophysiologyical (CEAP) classification, which was revised in 2004,^12^ and the latter was the venous clinical severity scoring (VCSS) which had been developed as a complementary to the first scheme in 2000^13^ and was revised in 2010.^14^ In CEAP, there were 8 classes designated as C0 (no visible or palpable signs of venous disease); C1 (telangiectasies or reticular veins); C2 (varicose veins); C3 (edema); C4a (pigmentation); C4b (lipodermatosclerosis); C5 (healed venous ulcer); and C6 (active venous ulcer). In VCSS, there were 10 questions that assessed the intensity of pain, varicose veins, venous edema, skin pigmentation, inflammation, induration, ulcers (number, duration and size), and use of compressive therapy. Each question had 4 possible answers (0: none, 1: mild, 2: moderate, and 3: severe). Points were added to produce a total score ranging between 0 and 30. Total score of CEAP ranged between 0 and 7. The clinical signs in only the more affected leg were considered while grading. Symptoms that could be associated with venous thrombosis, such as itching, cramps, and leg hair loss, were also assessed. Venous claudication was specifically sought using the Edinburgh questionnaire—the revised version of the Rose questionnaire.^15^ Severe PTS was defined as CEAP classes 4 through 6.^16^

The institutional review board of the Cerrahpaşa Medical School approved the study and all participants gave informed consent.

### Statistical Analysis

Continuous data were expressed as mean and standard deviation (SD). Data with non-normal distribution were expressed as median (interquartile range [IQR]). Comparisons between groups were made by Student *t* test for normal-distributed continuous variables. Continuous variables with non-normal distribution were compared using Mann–Whitney *U* test. Categorical variables were compared by the Pearson chi-square test or the Fisher exact test.

Hierarchical cluster analysis was used to identify patterns of venous disease. Tree dendograms that were derived using ϕ correlation coefficient were constituted to visualize cluster patterns in both patient and control groups.

We used univariate and multivariate binary logistic regressions to determine variables that were independently associated with having BS, with control group being accepted as the reference group. To examine relationships between various predictors and severe PTS and venous claudication among BS patients, we again used univariate and multivariate binary logistic regressions. Those variables that were found to be associated significantly with univariate analyses were then used to build multiple logistic regression models.

The odds ratios (ORs) with 95% confidence intervals (CIs) were calculated by binary logistic regression models (Enter). All tests were performed using SPSS for Windows, version 20.0, software (IBM SPSS Inc., Chicago, IL).

## RESULTS

### Demographic and Clinical Characteristics of the Study Population

We studied 78 (71 men, 7 women) BS and 50 (29 men, 21 women) controls with LEVT. As shown in Table 1, BS patients were more likely to be men (91% vs 58%) and had significantly earlier age of onset of thrombosis (32 ± 9 vs 41 ± 13; *P* < 0.001). Among the BS patients, the median disease duration of BS—from the fulfillment of the criteria to the time of the current study—was 12 (IQR 7–19) years. The median time between BS and thrombosis onset was calculated as 5 years (IQR 2–9 years). Thrombosis duration was calculated as median 5 (IQR 1–10) years for BS and median 7 (IQR 1.3–11.9) months for controls.

**TABLE 1:**
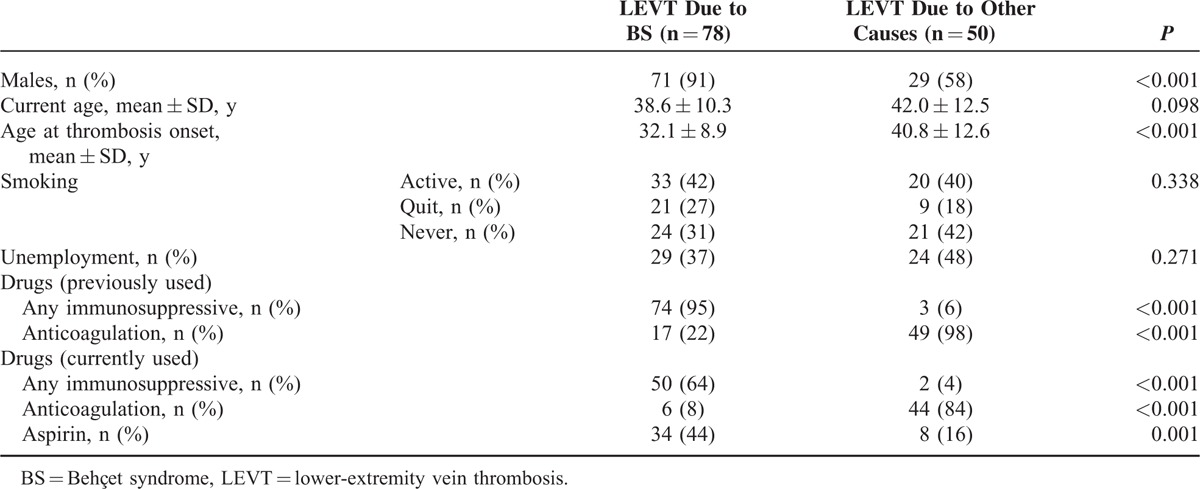
Demographic and Clinical Characteristics Among BS Patients and Non-BS Patients With LEVT

Smoking and unemployment were similar in frequency in both groups. These were also true when women were excluded.

Twenty-one (27 %) BS patients had concomitant one or more venous (n = 10) or arterial (n = 11) vascular involvement outside the legs. Venous thrombosis was found in the superior vena cava (n = 3), inferior vena cava (n = 6), dural sinus (n = 5), and hepatic veins (n = 3). Arterial aneurysms were observed in the pulmonary (n = 6), femoral (n = 2), popliteal (n = 1), carotid arteries (n = 1), and abdominal aorta (n = 1). Apart from vascular involvement, 23 (30 %) patients had joint, 38 (49 %) eye, and 1 patient had parenchymal neurological involvement.

Of the 50 controls with LEVT due to other causes, a possible cause for thrombotic tendency could be identified in 14, such as renal transplantation (n = 1), pregnancy (n = 5), long trip (n = 4), use of oral contraceptives (n = 1), traffic accident (n = 1), and postsurgical intervention (n = 2). Apart from these, 5 patients had been diagnosed with a chronic disease such as rheumatoid arthritis (n = 2), psoriatic arthritis (n = 1), ankylosing spondylitis (n = 1), and chronic hepatitis B (n = 1).

### Past and Present History of Drug Use

Seventy-four patients with BS (95 %) had been given at least 1 immunosuppressive, which was most commonly azathioprine (AZA) (n = 67, 91 %), after being diagnosed with thrombosis. Of these 74 patients, 44 (56 %) were also given corticosteroids and 17 (23 %) received anticoagulation. The remaining 4 (1 man, 3 women) BS patients had been given colchicine alone. On the contrary, 49 controls (98 %) had received anticoagulation.

At the time of the current study, BS patients were using most commonly AZA (64%) and aspirin (44%) less commonly, colchicine (18%), corticosteroids (13%), and anticoagulation (8%). The majority of the controls were, however, using anticoagulants (84%) and aspirin (16%). Similar number of patients were off-treatment in BS (14%) and in the control (8%) groups.

### Doppler USG Results

#### Current Doppler USG Lesions

Current Doppler USG detected complete recanalization depicting full recovery among 3 (4 %) BS patients, whereas 7 (14 %) among the controls (*P* = 0.047). In the remaining, there were similar number of thrombotic lesions (97% vs 95%), partial recanalization (91% vs 91%), and reflux (15% vs 19%). Thrombotic lesions were mostly in the form of chronic thrombosis in both groups (85% vs 81%). Current Doppler studies also showed collateral formation, which was significantly more common among BS patients (31% vs 12%; *P* = 0.02). Unlike thrombosis, recanalization, and reflux, which are seen in all types of veins, collaterals were specifically observed in the CFV, SFV, and VSM.

#### Unilateral/Bilateral Involvement

The first available Doppler USG had demonstrated that the majority in both groups had unilateral involvement; yet this was significantly more common among the controls (BS: 50/78 [64%] vs controls: 46/50 [92%]; *P* < 0.001). The current Doppler studies showed that less patients remained with unilateral involvement in both groups, but again this was significantly more common in the control group (BS: 35% vs controls: 61%; *P* = 0.007).

#### Anatomical Localization of Lower-extremity Vein Involvement

**FIGURE 1:**
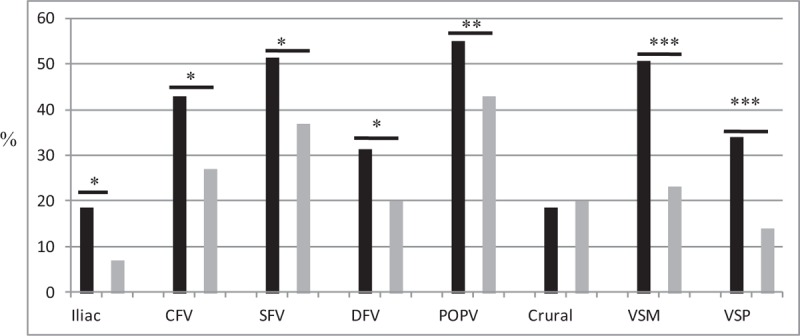
Distribution of veins among Behcet syndrome patients with LEVT (black bars) and controls with LEVT due to other causes (gray bars). ^∗^*P* < 0.05, ^∗∗^*P* < 0.005, ^∗∗∗^*P* < 0.001. CFV = common femoral vein, DFV = deep femoral vein, LEVT = lower extremity vein thrombosis, POPV = popliteal vein, SFV = superficial femoral vein, VSM = vena saphena magna, VSP = vena saphena parva.

Figure 1 shows the anatomical locations of the involved veins. Except the crural veins, all deep and superficial veins were significantly more likely to be involved among BS patients. Additionally, there were significantly more patients with both deep and superficial vein (VSM and/or VSP) involvement (n = 56, 72%), as compared with the controls (n = 20, 40%; *P* < 0.001). The total number of veins involved per patient was significantly higher among BS patients (6.05 ± 3.86 vs 3.82 ± 2.55; *P* = 0.004).

The probability of having iliac vein involvement in any case was calculated as 27% (95% CI 18–38) for BS and 12% (95% CI 6–24) for controls. In case of CFV involvement, this probability went up to 47% (95% CI 33–61) for BS and to 26% (95% CI 13–46) for controls.

### Variables Strongly Associated with Having BS

According to the multivariate logistic regression testing, variables associated with having BS were defined as being male (OR 6.8) and younger than ≤35 years of age at the onset of thrombosis (OR 4.1), and having bilateral femoral veins (CFV, SFV, and DFV) (OR 6.8) and VSM involvement (OR 4.0) (Table 2).

**TABLE 2:**
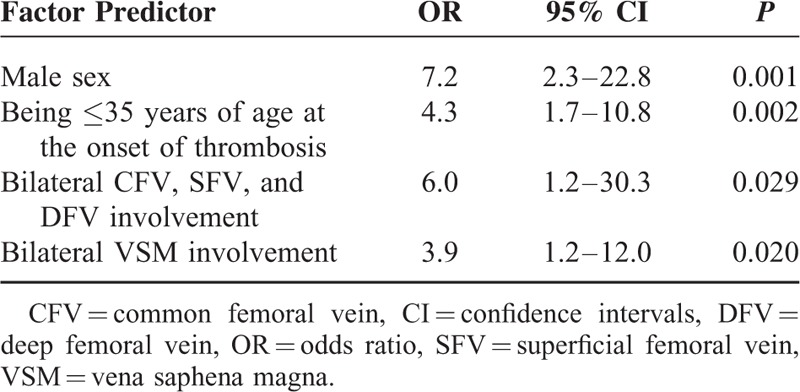
Multivariate Logistic Regression Testing for Variables Associated With Lower-extremity Vein Thrombosis Due to Behcet Syndrome

#### Cluster Analysis of Veins

**FIGURE 2:**
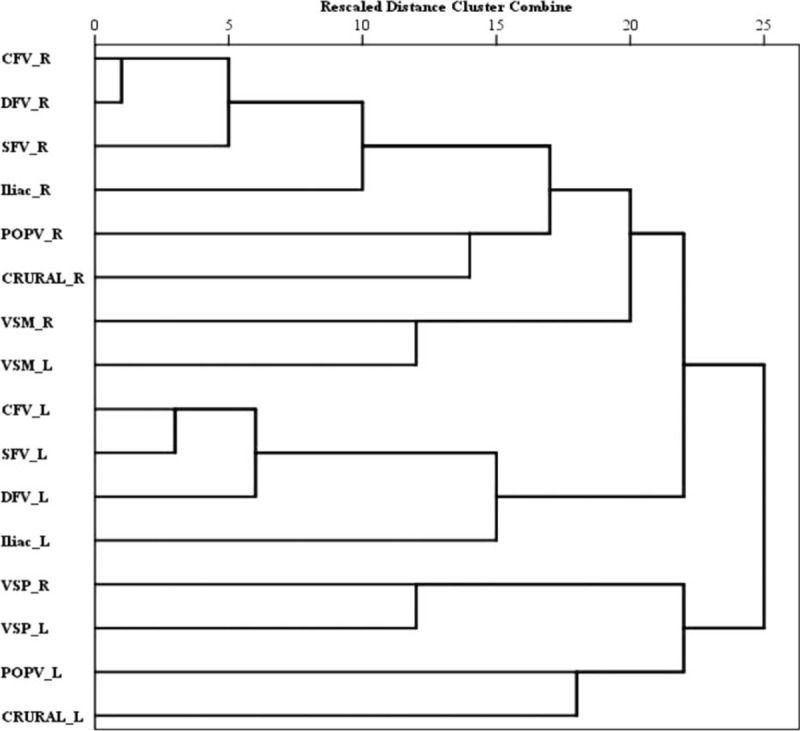
Dendogram derived using ϕ correlation coefficient for lower-extremity veins thrombosed in Behcet syndrome.

In BS, there was almost a symmetrical order in the cluster dendograms, in that venous involvement tended to be contiguous on either side (Fig. 2). CFV, DFV, and SFV, and further iliac vein formed a cluster on the right side. Later on, this cluster was joined by the cluster formed by PV and crural veins, again on the right side. This was similarly duplicated on the left side in a symmetrical pattern. On the contrary, superficial veins (VSM and VSP) clustered always in a symmetrical pattern.

**FIGURE 3:**
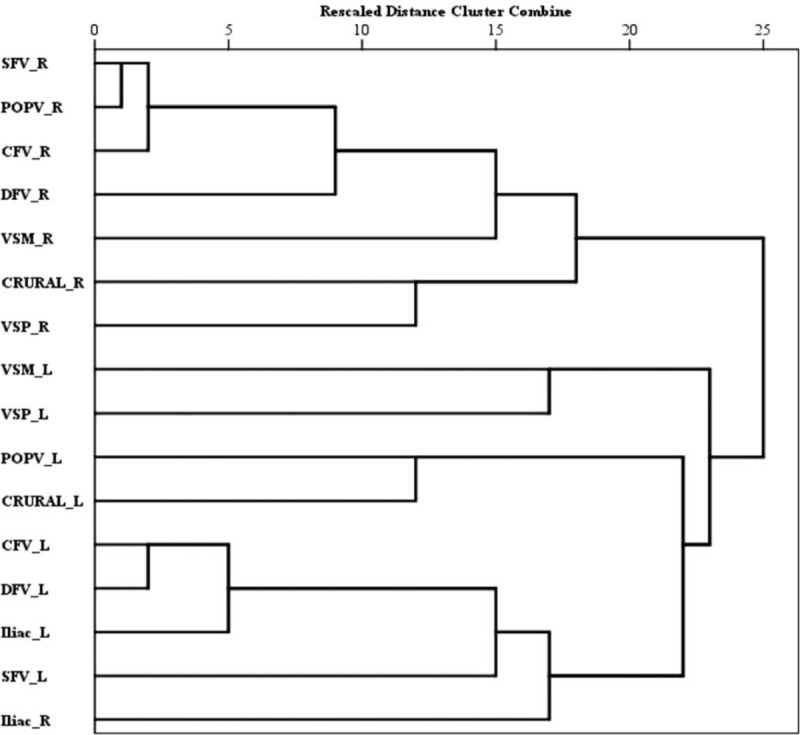
Dendogram derived using ϕ correlation coefficient for lower-extremity veins thrombosed in controls.

In the control group, however, the associations were disordered (Fig. 3).

### Clinical Assessment of Severity

Signs and symptoms assessed with the VCSS classification, CEAP grading, and the frequency of venous claudication are shown in Table 3.

**TABLE 3:**
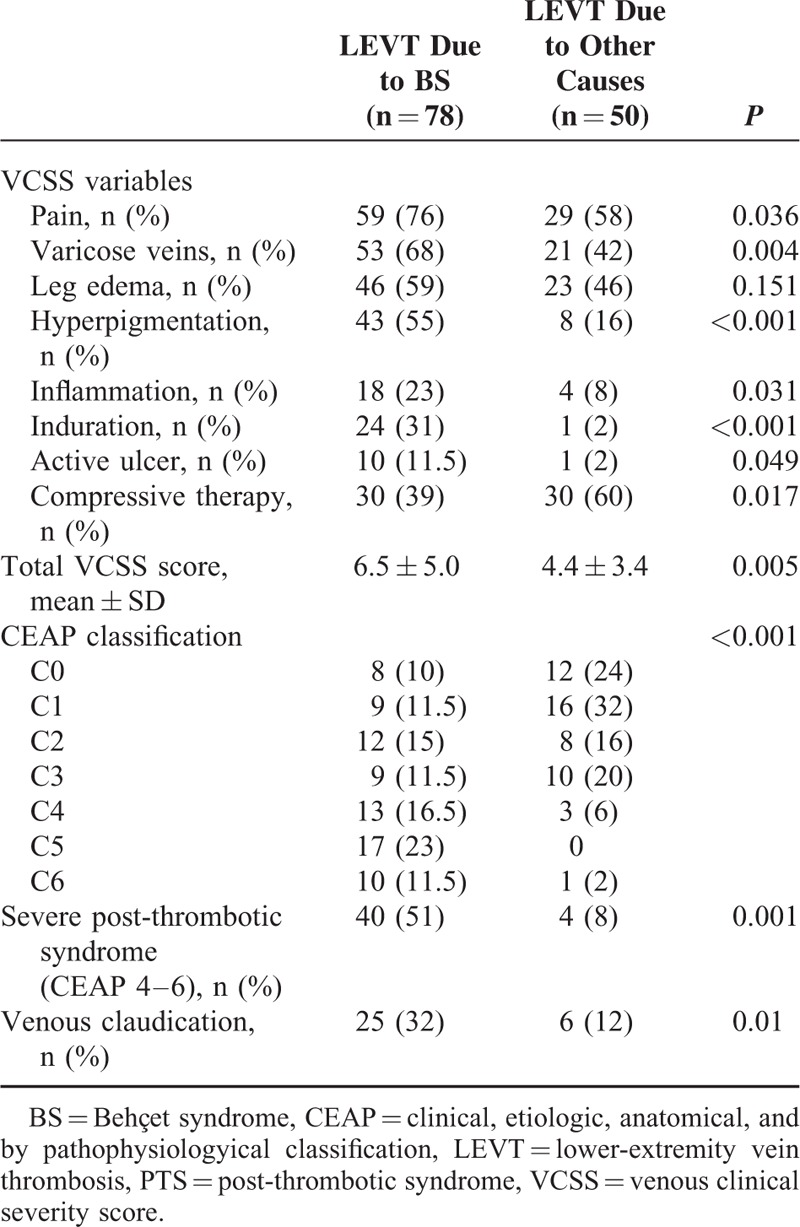
Signs and Symptoms Associated With VCSS, CEAP grading, severe PTS, and Venous Claudication

#### VCSS Classification

Almost all elements used to calculate the VCSS such as pain, varicose veins, hyperpigmentation, inflammation, induration, and active ulcers were significantly more frequent among the BS patients. Whereas leg edema was similar in frequency in both groups, elastic stocking use was significantly more frequent in the control group. Finally, total VCSS score was significantly higher in the BS group. Symptoms unrelated with the VCSS were also assessed: leg hair loss was more (35% vs 14%; *P* = 0.010), whereas cramping was less common (30% vs 50%; *P* = 0.019) among BS patients. The frequency of itching was comparable in both groups (35% vs 34%).

Venous clinical severity scoring and CEAP scores were significantly correlated with each other (Spearman *r* = 0.776, *P* < 0.0001).

#### CEAP Grading: Post-thrombotic Syndrome

Behcet syndrome patients had more severe CEAP grades, and the frequency of patients with severe PTS (CEAP ≥4) was significantly higher in the BS group (51% [40/78] vs 8% [4/50]; *P* < 0.0001). These were also true after adjusting for age and sex (data not shown). It has to be noted that thrombosis duration among BS patients (median 5 years) was substantially longer than that among controls (median 8 months). Therefore, to adjust for thrombosis duration in both groups, only those with thrombosis duration of ≤1 year were analysed. The frequency of PTS was again found to be significantly higher among BS patients (30% [5/17] vs 8% [3/40]; *P* = 0.045).

#### Venous Claudication

One-third of the patients with BS, whereas 12% of the controls complained from venous claudication (*P* = 0.01). This was also true when 4 BS patients with peripheral arterial aneurysms were excluded (23/74, 31% vs 6/50, 12%; *P* = 0.017). When only those with thrombosis duration of ≤1 year were analyzed, the frequency of venous claudication was again higher in BS, albeit not significant (24% [4/17] vs 12.5% [5/40]; *P* = 0.428).

#### Clinical and Ultrasonographic Parameters Associated with Severe PTS and Venous Claudication in BS

As shown in Table 4, having a thrombosis duration of more than ≥5 years (OR 3.3), having bilateral femoral vein (CFV, SFV, and DFV) involvement (OR 3.4), and using no anticoagulation along with immunosuppressive treatment when thrombosis was first diagnosed (OR 3.8) were found to be associated independently with severe PTS.

**TABLE 4:**
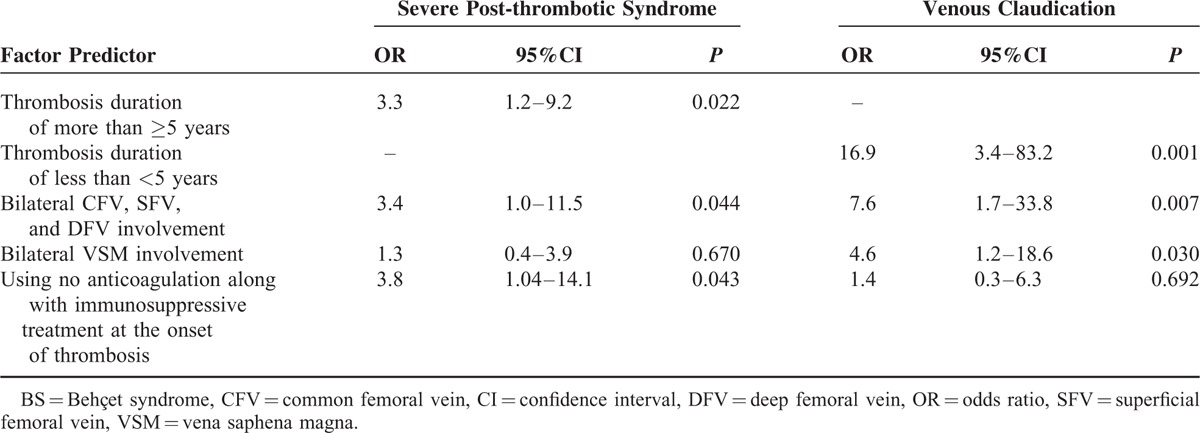
Multivariate Logistic Regression Analyses for Variables Associated With Severe Post-thrombotic Syndrome and Claudication in BS

Multiple regression analyses revealed that venous claudication was significantly associated with having thrombosis duration of less than 5 years, and bilateral femoral vein (CFV, SFV, and DFV) involvement and bilateral VSM involvement (Table 4). Using anticoagulation did not affect the presence or absence of claudication.

The associates of severity could not be calculated in the control group, since there were a few patients with severe PTS or venous claudication.

#### Differences Between Sexes

We also noted that the female patients with BS had significantly less severe VCSS (2.4 ± 2.2 vs 6.9 ± 5.1; *P* = 0.023) and CEAP grades (1.7 ± 2.1 vs 3.9 ± 2.3; *P* = 0.018), and had significantly lower number of thrombotic veins (2.4 ± 2.7 vs 4.8 ± 2.5; *P* = 0.020). On the contrary, among controls, men and women were similar with regard to severity scores (VCSS: 4.3 ± 4.0 vs 4.5 ± 2.2, respectively; *P* = 0.87; CEAP: 1.7 ± 1.7 vs 1.6 ± 1.2, respectively; *P* = 0.79) and number of involved veins (3.1 ± 2.0 vs 3.5 ± 2.6; *P* = 0.48).

## DISCUSSION

In this cross-sectional study, we evaluated the pattern of venous distribution and the clinical impact of LEVT associated with BS and controls. With a controlled study, we are now able to describe a list of clinical and radiological characteristics of LEVT that might be rather specific for BS. Being male and having young age of onset at thrombosis, and having bilateral involvement of deep proximal (CFV, SFV, and DFV) and superficial veins (VSM) were found to be strongly associated with having LEVT associated with BS.

Certain well described features of LEVT due to BS were also noted in this study: LEVT in BS usually occurs within 5 years of disease onset, affects mostly proximal part of the lower extremity venous system similar to that seen in the general population, and is frequently associated with other types of vascular disease.^3,17–21^

Our cluster analyses showed that the pattern of venous involvement in BS was contiguous and symmetric in both legs. Starting usually from the CFV, it progresses to both the proximal and the distal veins, affecting deep and superficial veins together, with less affinity for crural veins. In the control patients, however, thrombosis was most commonly unilateral; the combination of deep and superficial vein involvement was less common: consequently, the total number of involved veins was significantly less and the pattern of venous distribution was disorganized.

In addition to the contiguous and symmetric pattern, we observed that there was less complete recanalization and more frequent collateral formation among BS patients compared with controls, suggesting a chronic relapsing course and probably related to that a more obliterative and diffuse thrombotic activity. In fact, it could be quite possible that all these distinctive ultrasonographic and clinical characteristics that we find in these patients may simply result from the relapsing course, unique to this syndrome. Moreover, as we and others have previously reported, there is an increased tendency to form collaterals in both venous and arterial side in BS.^22–24^

In line with the radiological indication of a severe disease, clinical elements of the chronic venous disease were also commonly present in BS. About half of the BS patients showed signs of severe PTS, and one-third suffered from venous claudication, whereas these complications were present in 8% and 12%, respectively, in the control group. It is known that PTS may develop in 20% to 50% of patients after LEVT in the general population.^7–10^ However, severe PTS was seen in less than 5% in 387 patients with acute DVT, in a 24-month prospective survey.^25^ In another survey including 355 consecutive patients, it was less than 10% after 8 years of follow-up.^26^

Determinants of PTS in the general population were venous thrombosis of the common femoral or iliac vein, a higher body mass index, previous ipsilateral venous thrombosis, older age, and female sex.^10^ In our survey, however, while bilateral femoral vein involvement was equally important for PTS in BS, quiet different from the general population, young age of onset and male sex were the other determinants for PTS.

We manage BS patients with LEVT differently than those with other causes: immunosuppressive agents are the mainstay of treatment, whereas anticoagulation in addition to immunosuppressives is seldom used, as was the case among the patients we describe here. We have the following reasons to support this approach:

1. The information gathered from our radiologists who do the USG studies routinely and that from the vascular surgeons who routinely perform aneurysms or occlusion operations tell us that thrombus in BS develops as a complication to underlying vasculitis; with relapses, the thrombotic vein evolves into a dense, fibrotic structure, posing little risk for emboli.
2. Pulmonary embolism is indeed extremely rare in post mortem series^27^ and according to our group's clinical experience of almost 40 years. In a very recent thorax computed tomography (CT) study among 49 BS patients with new LEVT, we did not see a single scan showing pulmonary emboli.^28^ On the contrary, pulmonary artery thrombus (PAT) that we usually see in BS develops in situ and is manifested as a clinical form of pulmonary artery involvement similar to pulmonary artery aneurysms that requires intensive immunosuppression.^29^ Additionally, as we have previously demonstrated before,^29^ V/Q scintigraphic changes persist in PAT due to BS in contrast to what is observed in pulmonary emboli in which these changes tend to resolve.
3. Considering the strong association between pulmonary artery involvement and LEVT, there is a risk of massive hemoptysis with anticoagulation. Because of the intensive collateral formation, this risk may still persist during the remission period.
4. Finally, a recent large survey and two others found no additional benefit in terms of thrombotic relapse rate of anticoagulation used in combination with immunosuppressives, compared with immunosuppressives alone.^20,30,31^

On the other hand, in the current survey, we were surprised to note that additional anticoagulation was associated with less PTS. While this finding is based on a small subgroup and is subject to limitations of a retrospective design, such as the possibility of those patients having been started on anticoagulants by the referring physicians because of PTS, about which we do not have formal data, it surely points to the long time voiced need of a properly conducted randomized controlled trial of the role of anticoagulation in venous disease in BS. A further consideration is that some elements of the PTS observed in BS might not just be the simple result of the occlusion of the medium to large veins in BS. A possible contribution of the smaller vessels (both venules and arterioles) in the skin may also play a role in the formation of PTS associated with BS. We are unaware of any related published work.

Finally, venous claudication, we believe, is as much as important as PTS; however, it is being underestimated while evaluating the clinical impact of chronic venous disease. In a recent study, including an almost different cohort, we used a standardized treadmill exercise challenge, similar to that used for the investigation of peripheral arterial disease to evaluate the frequency and severity of venous claudication among BS patients with LEVT and age, and sex-matched healthy controls.^6^ While 34% of the BS patients with LEVT described venous claudication according to the questionnaire, 21% complained from claudication during the treadmill test and 10% had to stop the treadmill before the scheduled time. Determinants of venous claudication in the current survey were bilateral femoral involvement, and superficial vein thrombosis and shorter duration of thrombosis. The latter findings suggest that this symptom is somewhat an early symptom and that may disappear or be relieved in time probably once collaterals are formed. It has to be noted that unlike PTS, the frequency of venous claudication did not differ between those who received anticoagulation and those who did not. As LEVT occurs mostly among young men in BS—the potentially productive and active fraction of the population—the clinical and social consequences of this problem such as unemployment, working disability, impairment of quality of life, and depressive mood disorders would be inevitable. We suggest that venous claudication should be included in the routine clinical assessment of vascular disease in BS.

Our study had some limitations. We did not look at the interobserver and intraobserver variability of Doppler USG measurements. We did not study those patients and controls with only venous insufficiency. Doppler USG could not be an optimum tool for iliac vein evaluation in some patients. Finally, we could not match BS patients and controls for duration of thrombosis. On the contrary, we now think that this would be quite difficult, given the obvious differences of disease course between the two forms of LEVT: the considerably shorter thrombosis duration observed in the control group (median 7 months), contrasted with that observed among the BS patients (median 5 years), suggesting probably that idiopathic LEVT do not relapse and often is self-limited. In this setting, patients with other chronic inflammatory diseases such as systemic lupus erythematosus, antiphospholipid syndrome, or Anti-neutrophil cytoplasmic antibodies-associated vasculitides might have been desirable as additional and perhaps more appropriate control groups; however, LEVT is relatively rare among these patients. As there have been no well defined associated coagulation abnormalities in BS,^32–35^ we do not routinely examine thrombophylic factors in our patients. This omission could also be a limitation, especially considering those patients with extensive recurrent LEVT.

## CONCLUSIONS

Lower-extremity vein thrombosis associated with BS has some distinctive demographic and clinical characteristics when compared to LEVT due to other causes or idiopathic LEVT. It occurs mostly among young men, affects deep and superficial lower-extremity veins often together, and tends to be contiguous and bilateral. There is a more severe disease course with half of the patients suffering from severe PTS and one-third from venous claudication.
